# MRI-guided microwave ablation and albumin-bound paclitaxel for lung tumors: Initial experience

**DOI:** 10.3389/fbioe.2022.1011753

**Published:** 2022-11-03

**Authors:** Xiaokang Shen, TianMing Chen, Nianlong Liu, Bo Yang, GuoDong Feng, Pengcheng Yu, Chuanfei Zhan, Na Yin, YuHuang Wang, Bin Huang, Shilin Chen

**Affiliations:** ^1^ Department of Thoracic Surgery, Jiangsu Cancer Hospital, Nanjing, China; ^2^ Department of Cardiothoracic Surgery, Nanjing First Hospital, Nanjing Medical University, Nanjing, China; ^3^ Department of General Surgery, Nanjing Drum Tower Hospital, The Affiliated Hospital of Nanjing University Medical School, Nanjing, China; ^4^ Department of Thoracic Surgery, The Affiliated Cancer Hospital of Nanjing Medical University, Jiangsu Cancer Hospital, Jiangsu Institute of Cancer Research, Nanjing, Jiangsu, China; ^5^ Department of Medical Imaging, Jiangsu Cancer Hospital, Jiangsu Institute of Cancer Research, The Affiliated Cancer Hospital of Nanjing Medical University, Nanjing, Jiangsu, China; ^6^ Department of Interventional Therapy, Jiangsu Institute of Cancer Research, The Affiliated Cancer Hospital of Nanjing Medical University, Nanjing, China; ^7^ The Comprehensive Cancer Center of Nanjing Drum Tower Hospital, The Affiliated Hospital of Nanjing University Medical School, Clinical Cancer Institute of Nanjing University, Nanjing, China; ^8^ The Comprehensive Cancer Centre of Nanjing Drum Tower Hospital, Clinical College of Nanjing Medical University and Clinical College of Traditional Chinese and Western Medicine, Nanjing University of Chinese Medicine, Nanjing Drum Tower Hospital, Medical School of Southeast University, Nanjing, China

**Keywords:** albumin-bound paclitaxel, magnetic resonance imaging, microwave ablation, non-small cell lung cancer, safety and efficiency

## Abstract

Magnetic resonance-guided microwave ablation (MRI-guided MWA) is a new, minimally invasive ablation method for cancer. This study sought to analyze the clinical value of MRI-guided MWA in non-small cell lung cancer (NSCLC). We compared the precision, efficiency, and clinical efficacy of treatment in patients who underwent MRI-guided MWA or computed tomography (CT)-guided microwave ablation (CT-guided MWA). Propensity score matching was used on the prospective cohort (MRI-MWA group, *n* = 45) and the retrospective observational cohort (CT-MWA group, *n* = 305). To evaluate the advantages and efficacy of MRI-guided MWA, data including the accuracy of needle placement, scan duration, ablation time, total operation time, length of hospital stay, progression-free survival (PFS), and overall survival (OS) were collected and compared between the two groups. The mean number of machine scans required to adjust the needle position was 7.62 ± 1.69 (range 4–12) for the MRI-MWA group and 9.64 ± 2.14 (range 5–16) for the CT-MWA group (*p* < 0.001). The mean time for antenna placement was comparable between the MRI and CT groups (54.41 ± 12.32 min and 53.03 ± 11.29 min, *p* = 0.607). The microwave ablation time of the two groups was significantly different (7.62 ± 2.65 min and 9.41 ± 2.86 min, *p* = 0.017), while the overall procedure time was comparable (91.28 ± 16.69 min vs. 93.41 ± 16.03 min, *p* = 0.568). The overall complication rate in the MRI-MWA group was significantly lower than in the CT-MWA group (12% vs. 51%, *p* = 0.185). The median time to progression was longer in the MRI-MWA group than in the CT-MWA group (11 months [95% CI 10.24–11.75] vs. 9 months [95% CI 8.00–9.99], *p* = 0.0003; hazard ratio 0.3690 [95% CI 0.2159–0.6306]). OS was comparable in both groups (MRI group 26.0 months [95% CI 25.022–26.978] vs. CT group 23.0 months [95% CI 18.646–27.354], *p* = 0.18). This study provides hitherto-undocumented evidence of the clinical effects of MRI-guided MWA on patients with NSCLC and determines the relative safety and efficiency of MRI- and CT-guided MWA.

## Introduction

Percutaneous thermal ablation under imaging guidance is a minimally invasive technique used for the palliative treatment of nonoperative patients with non-small cell lung cancer (NSCLC) and pulmonary metastasis (Dupuy, 2011). As a heat-based ablation technique, microwave ablation destroys tissues using an electromagnetic field (typically 50–60 W), and the feasibility and efficacy of this approach have been extensively validated (Goldberg et al., 2000; Vietti Violi et al., 2018). Computed tomography (CT) and ultrasound are the most widely used traditional imaging modalities for ablation guidance. It is widely acknowledged that CT-guided treatment of lung cancer via microwave ablation entails significant limitations, such as inaccurate ablation-needle puncturing and inaccurate assessment of the ablation boundary, which affect its therapeutic efficacy to a certain extent (Lu et al., 2012; An et al., 2022). Magnetic resonance imaging (MRI) has limited value for diagnosing and treating lung diseases due to motion artifacts and low signals (Boiselle et al., 2013; Ciet et al., 2015). Over the years, MRI has gained significant momentum with the emergence of new imaging techniques such as multi-channel MRI, systems with strong gradients, and innovative pulse-train techniques for parallel imaging (Puderbach et al., 2007). MRI brings many advantages, including arbitrary direction imaging and high tissue resolution, and can identify changes in the ablation zone during the entire process of ablation, which is useful for monitoring changes in the ablation region (Li et al., 2017; Shono et al., 2020; Shen et al., 2021). However, few studies have assessed the clinical utility and economic value of MRI-guided MWA. To our knowledge, this is the first study to evaluate the accuracy and applicability of MRI-guided MWA therapy.

## Materials and methods

### Patients and tumor criteria

This prospective study was approved by the Ethics Committee of Jiangsu Provincial Hospital, affiliated with Nanjing Medical University. All patients included in the study provided written informed consent.

**FIGURE 1:**
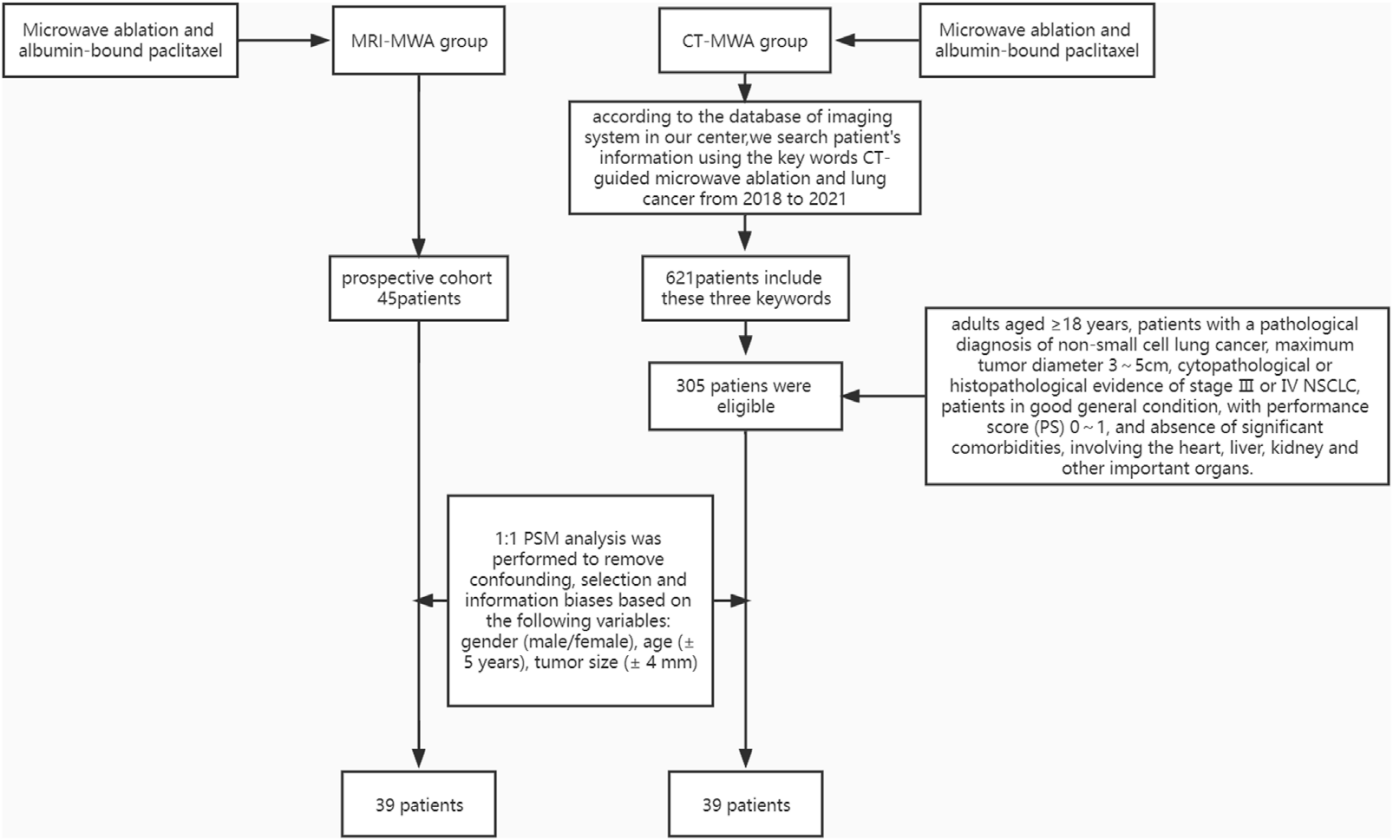
Screening process for eligible patients.

Subjects in this study included a prospective cohort (MRI-MWA group, *n* = 45) with lung tumors treated with MRI-guided MWA combined with chemotherapy from February 2018 to August 2021 and a retrospective, observational cohort (*n* = 305) of consecutive patients who underwent CT-guided MWA combined with chemotherapy (CT-MWA group) at our hospital between January 2018 and June 2021. One-to-one propensity score matching (PSM) analysis was performed to remove confounding, selection, and information biases based on the following variables: gender (male/female), age (± 5 years), tumor size (± 4 mm), tumor location (peripheral third of lung, middle third of lung, or inner third of lung), histology, and TNM stage, yielding 39 patients in the MRI-MWA group and 39 patients in the CT-MWA group (Figure1).

For inclusion in the study, patients were required to be adults aged ≥ 18 years; to have a pathological diagnosis of NSCLC, a maximum tumor diameter of 3∼5 cm, and cytopathological or histopathological evidence of stage III or IV NSCLC; and to be patients in good general condition, with performance scores of 0–1 and an absence of significant comorbidities involving the heart, liver, kidney, and other major organs.

The exclusion criteria removed patients who could not tolerate additional surgery due to severe cardiopulmonary comorbidities and those in poor physical condition, such as those with insufficient pulmonary function.

## Microwave ablation protocol

### MRI scanning program and parameters

The Philips Ingenia TM 3.0T system (Philips Medical Systems, Amsterdam, Netherlands) was used to guide and to monitor the microwave ablation procedures with a 16-channel phased array coil. MRI-guided MWA was performed using an ECO-100A MWA system (ECO Medical Instruments Co., Ltd. Nanjing, China; CFDA Certificated No. 20173251268). Moreover, the cooled-shaft ceramic antenna (ECO-100CI8) was made of nonmagnetic ceramic material. The scanning parameters used are shown in Table 1.

**TABLE 1:** Summary of sequence parameters used for imaging.

Sequence	T_1_-TFE	T_2_-TSE	DWI
TR (ms)	10	1129	749
TE (ms)	2.3 ACT	80	70
Time(s)	14	17	36
REC Voxel (mm)	0.93/0.93/4.00	0.78/0.78/4.00	1.25/125/4.00
Flip Angle (deg)	15	125	90
FOV(mm)	400 × 330	400 × 330	400 × 330
Slice thickness (mm)	4.0	4.0	4.0

### CT scanning program and parameters

All lung microwave ablations were performed using CT fluoroscopic guidance (Somatom Sensation 64; Siemens, Erlangen, Germany) with the following parameters: 5-mm collimation, 30 mAs, 120 kV, and 5-mm section thickness. The microwave system consisted of the ECO-100A MWA delivery system (ECO Medical Instruments Co., Ltd. Nanjing, China; CFDA Certificated No. 20173251268), and the cooled-shaft antenna (ECO-100AI13) was made of composite metal materials.

### Ablation procedure

The microwave ablation procedure was performed under aseptic conditions by three interventional radiologists with more than eight years of experience in thoracic intervention, a technician, and two experienced radiologists. Codeine phosphate tablets were used to relieve cough (30 mg per patient). Local anesthesia (1% lidocaine) was provided to ensure patients could tolerate the ablation procedure. Electrophysiological monitoring and pulse oximetry were conducted and recorded throughout the procedure. Power was generally 40–60 W, and the treatment duration was 8–16 min. At the end of treatment, patients were instructed to hold their breath. A 20-W power-track ablation was performed immediately to avoid tumor implantation metastasis. After therapy, MRI/CT scanning was performed to observe the incidence of complications and to evaluate treatment outcomes in order to determine whether additional ablation was needed.

### MRI-guided MWA

**FIGURE 2:**
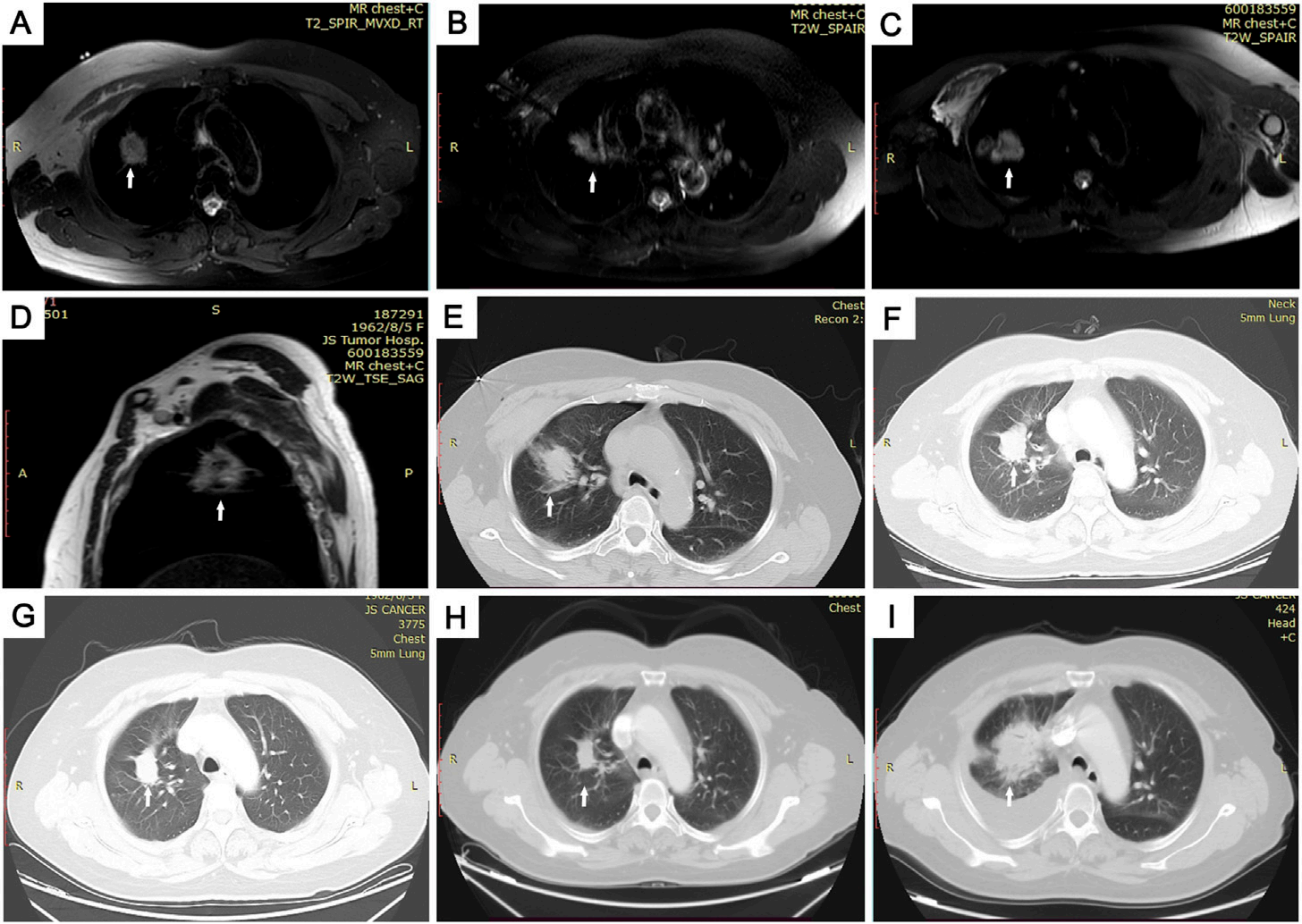
Images of the MRI-guided MWA procedure and follow-up in 59-year-old woman with pulmonary adenocarcinoma in superior lobe of right lung. **(A)** Skin markers were applied to locate the needle insertion site under magnetic resonance imaging (MRI) guidance. **(B)** Guided by magnetic-resonance T1WI image, 16G-ablation antenna was punctured to the center of the tumor lesion. **(C–E)** After two 8-min ablation cycles, we achieved a satisfactory ablation area, which is clearly seen in axial- **(C)** and sagittal- **(D)** phase images, and a large area of ground glass covers the entire tumor on the lung window of CT **I**. **(F–I)** Computed tomography (CT) images of follow-up. Follow-up CT scans at 3 **(F)**, 6 **(G)**, and 12 **(H)** months show significantly shrinking, and follow-up CT scan at 21 months **(I)** showed the tumor had increased in size.

**FIGURE 3:**
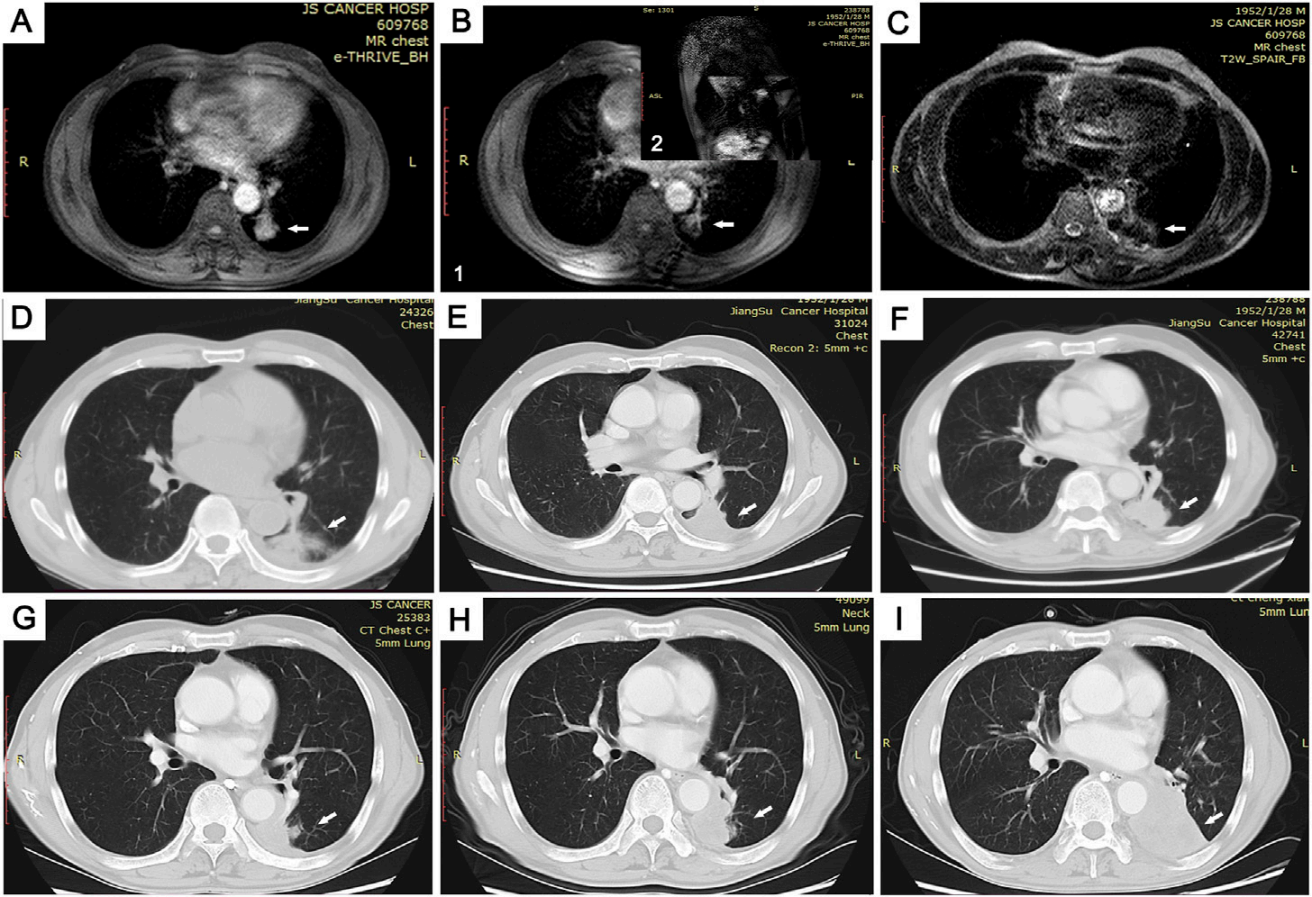
Images of the MRI-guided MWA procedure and follow-up in 69-year-old man with pulmonary adenocarcinoma in inferior lobe of left lung. **(A)** Tumor lesion seen on MRI immediately prior to MWA. **(B)** Combined with axial (1) and sagittal (2) MRI scans, the ablation needle reached the tumor site **(C,D)**. By comparing T2WI MRI scan images **(C)** and lung window CT images **(D)**, we found MRI to be more accurate in the assessment of microwave-ablation boundaries. (**E-I)** Follow-up CT scans at 3 **(E)**, 6 **(F)**, 12 **(G)**, and 20 **(H)** months showed no change in the GGO size. At the 26-month follow-up **(I)**, the tumor size was increased.

**FIGURE 4:**
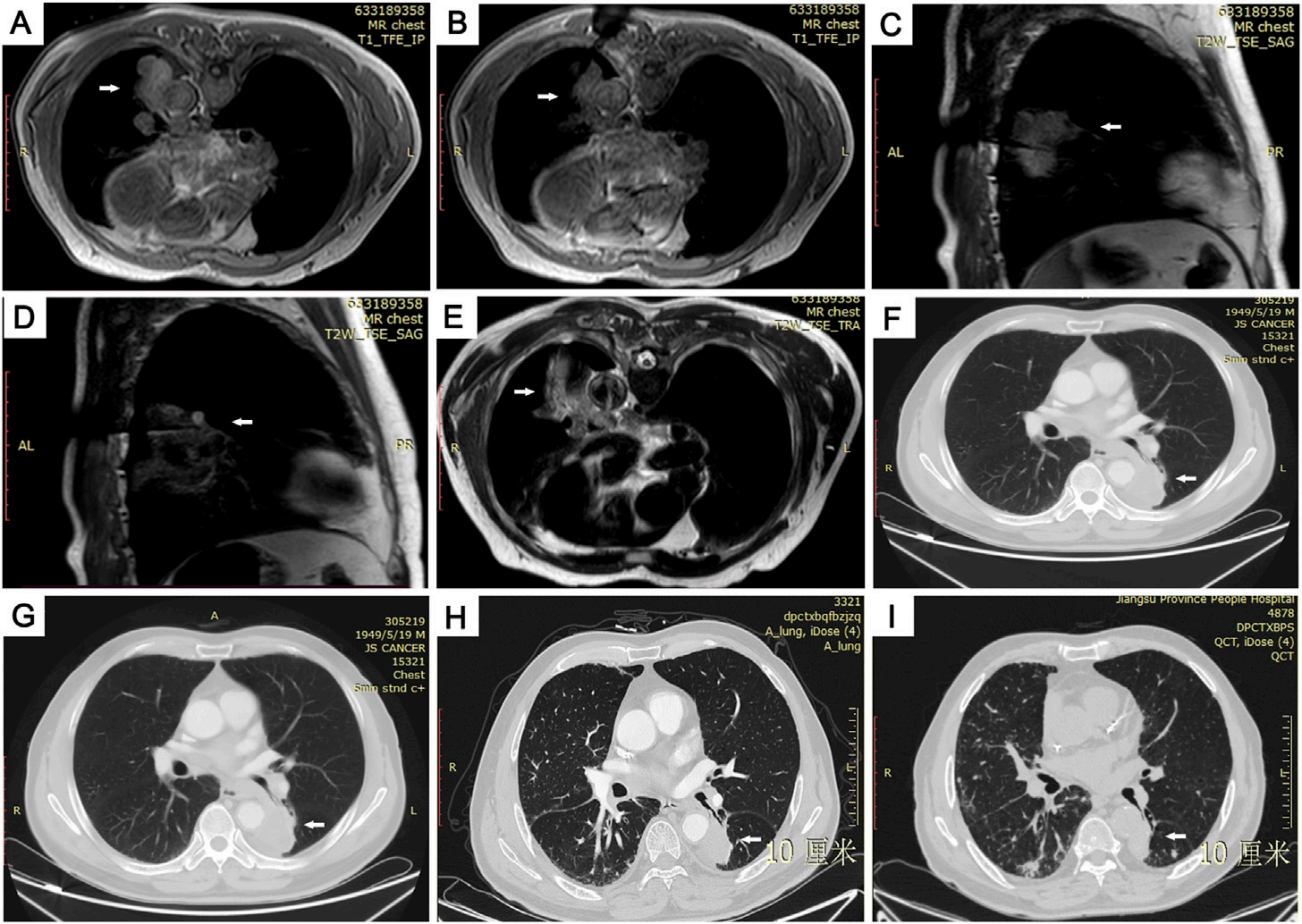
Images from 72-year-old man with 50 × 36 mm primary pulmonary adenocarcinoma in lower lobe of left lung. **(A)** Preoperative MRI scan and external puncture site location. **(B)** Ablation needle puncture reached the center of the lesion. **(C,D)** Due to the large lesion, a second ablation was performed by adjusting the ablation needle in real time under the sagittal image. **(E)** T2WI MRI scan immediately after the operation showing the surrounding ring-shaped high-signal thermal-injury response zone completely covered the ablation area with low signal and was well demarcated from the adjacent abdominal aorta. **(F–I)** Follow-up CT images showing the ablative zone was gradually shrinking at postoperative months 3 **(F)** 6 **(G)** 12 **(H)** and 16 **(I)**.

Multiple cod liver oil capsules were used as skin markers and were placed on the skin surface to determine the needle insertion site under MRI. The T1 and T2 sequences were combined to locate the puncture point and plan the preoperative puncture route. The whole-lung scan was performed on T2WI. Then, a breath-hold T1WI scan of the target region was performed. The microwave antenna has a low signal on the image. During the insertion process, horizontal, coronal, and sagittal MRI scanning was conducted during T1WI/TFE, providing a three-dimensional spatial relationship between the ablation needle and the tumor lesion, especially for irregular lesions. A vascular flow void on T2WI ensured that the puncture-ablation needle could effectively avoid important pulmonary vessels and prevent bleeding complications. T2WI/TSE, T1WI/TFE, and DWI sequences were scanned during MRI-guided ablation, and MWA dynamic intraoperative scans monitored changes in the MRI signals in the ablation foci. On T2WI, a low signal was found in the center of the ablated tumor, and the high signal area of the primary tumor disappeared and was surrounded by hyperemia and edema with a high signal, which was 5–10 mm beyond the primary tumor area, indicating successful ablation (Figure 2–4).

### CT-guided MWA

CT scans were performed to determine the puncture point as well as the angle and depth of the needle. Scans were repeated until the ablation needle reached the center of the tumor. The effect of ablation was evaluated by observing the change in lesion density on CT imaging. After ablation, the tumor lesions showed extensive high density with features such as the “honeycomb sign” and the “bubble sign.”

## Chemotherapy protocol

Three courses of albumin-bound paclitaxel combined with carboplatin were used after microwave ablation. Albumin-bound paclitaxel was used at a dose of 125 mg/m^2^ (injected for over 30 min). The drug was administered every three weeks on days 1 and 8. Cisplatin was administered at 75 mg/m^2^ every 3 weeks on day 1 (injected for over 60 min). The original protocol, when effective, was maintained for three cycles.

## Post-ablation follow-up

A chest X-ray or CT scan was performed after 24–48 h to identify any complications. Conservative treatment was adopted if the patient had developed a small asymptomatic pneumothorax or pleural effusion without significant complications. Follow-up CT was performed at 1-, 3-, 6-, and 12-month intervals after the initial ablation session using a multidetector-row helical CT scanner. Contrast-enhanced chest CT images were used to evaluate outcomes.

## Outcome evaluation and statistical analysis

The primary outcomes were the accuracy of needle positioning and the efficiency and safety of ablation, as guided by the two imaging methods.

The accuracy of ablation-needle puncture was defined as the targeting duration and number of scans required for ablation-needle placement.

The ablation efficiency included the microwave ablation time (min) and overall procedure time (min). The overall procedure time was determined as the time between the patient’s arrival in the CT or MRI room and the time of their departure. In most cases, less than two ablation cycles were required, ranging from 4 to 8 min. Ablation cycle duration depended on lesion size and power output.

Safety was assessed based on the presence of treatment-related complications, defined according to the Society of Interventional Radiology standard (Ahmed et al., 2014). Secondary outcomes consisted of indicators for evaluating curative effects, including the frequency of residual unablated tumors at 1 month after ablation, the median time to local tumor progression (PFS), and overall survival (OS) at 3 years.

Improved criteria from the Response Evaluation Criteria in Solid Tumors guidelines were used to evaluate the local tumor progression of patients at 3 years of follow-up (Watanabe et al., 2006; Nishino et al., 2010).

The variables were analyzed by SPSS 16.0 software. The means and standard deviations were determined for measurement data and were compared using the independent samples *t*-test or Wilcoxon rank-sum test. The χ2 test or Fisher’s exact test was used to compare categorical variables. The Kaplan–Meier method and log-rank test were applied to compare progression-free survival (PFS) and OS between groups. A *p*-value less than 0.05 was considered statistically significant.

## Results

Seventy-eight patients with lung cancer who underwent microwave ablation were included in this study. Patient demographics at baseline did not differ between the MRI and CT groups (*p* > 0.05; Table 2).

**TABLE 2:** Clinical characteristics of patients undergoing MRI-guided microwave ablation (MRI-MWA group) or CT-guided microwave ablation (CT-MWA group) to treat pulmonary malignancies.

Characteristic	Total patients (*n* = 78)	MRI-MWA group (*n* = 39)	CT-MWA group (*n* = 39)	χ^2^ value/*t*-value	*p*-value
Age	62.47 ± 8.61	61.89 ± 9.09	62.71 ± 8.18	0.188	0.666
Gender					
Male	48	23	25	0.217	0.642
Female	30	16	14		
Tumor size	7.77 ± 7.59	8.15 ± 8.64	7.39 ± 6.47	0.189	0.665
Location				0.665	0.717
Peripheral	39	18	21		
Middle	29	15	14		
Inner	10	6	4		
Pathology					
Adenocarcinoma	51	27	24	0.510	0.475
Non-adenocarcinoma	27	12	15		
TNM stage				0.228	0.892
IIIa	31	16	15		
IIIb	19	10	9		
IV	28	13	15		

Note—Data are means ±standard deviations or medians with interquartile ranges in parentheses for continuous variables.

There was no difference in the characteristics of the lesions, such as tumor size and depth. The mean number of scans required to adjust needle position was significantly less in the MRI group than in the CT group [7.62 ± 1.69 (range 4–12) vs. 9.64 ± 2.14 (range 5–16), *p* < 0.001]. The mean duration of antenna placement was comparable between the MRI and CT groups (54.41 ± 12.32 min and 53.03 ± 11.29 min, *p* = 0.607).

Microwave ablation duration was significantly different between the two groups (7.97 ± 2.311 min and 9.41 ± 2.86 min, *p* = 0.017).

The overall procedure duration was comparable between the MRI and CT groups (91.28 ± 16.69 min and 93.41 ± 16.03 min, *p* = 0.568) (Table 3).

**TABLE 3:** Comparison of observational data in both study groups.

Parameter	MRI-MWA group (*n* = 39)	CT-MWA group (*n* = 39)	t-value	*p*-value
Number of machine scans for adjusting needle position	7.62 ± 1.69	9.64 ± 2.14	21.396	<0.001
Time for antenna placement (min)	54.41 ± 12.32	53.03 ± 11.29	0.267	0.607
Microwave ablation time (min)	7.62 ± 2.65	9.41 ± 2.86	8.251	0.005
Total procedure time (min)	91.28 ± 16.69	93.41 ± 16.03	0.330	0.568
Residual unablated tumors	3 (7.69%)	4 (10.2%)	0.157	0.692

Note.—Unless otherwise specified, data are presented as mean with the range in parentheses.

Four major complications related to the ablation procedure were observed within 30 days in the MRI- and CT-MWA groups, including hemoptysis (*n* = 1 and *n* = 4, respectively), pleural effusion (*n* = 3 and *n* = 4, respectively), pneumothorax (*n* = 6 and *n* = 5, respectively), and cavitation of the ablation zone (*n* = 2 and *n* = 7, respectively) (Table 4). The overall complication rates were 12% and 51% in the MRI- and CT-MWA groups, respectively (*p* = 0.185).

**TABLE 4:** Complications.

Complication	MRI-MWA group (*n* = 39)	CT-MWA group (*n* = 39)	χ^2^ value	*p*-value
	12 (30%)	20 (51%)	6.203	0.185
Hemorrhage	1	4		
Pleural effusion	3	4		
Pneumothorax	6	5		
Cavitation of the ablation zone	2	7		

Note.—Data are numbers of patients with percentages in parentheses for categorical variables.

**FIGURE 5:**
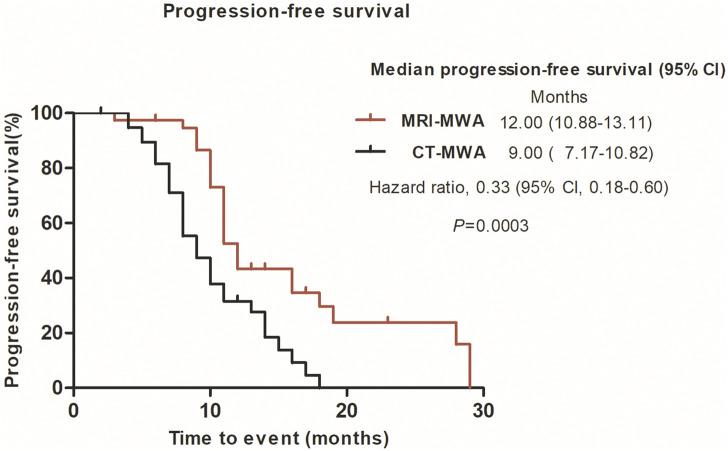
Graphs show Kaplan–Meier survival estimates for progression-free survival between MRI-MWA group and CT-MWA group.

The median time to progression was significantly longer in the MRI-MWA group than in the CT-MWA group (11 months [95% CI 10.24–11.75] vs. 9 months [95% CI 8.00–9.99], *p* = 0.0003; hazard ratio 0.3690 [95% CI 0.2159–0.6306]). As shown in Figure 5, Kaplan–Meier analysis revealed a significant difference in PFS between both groups.

**FIGURE 6:**
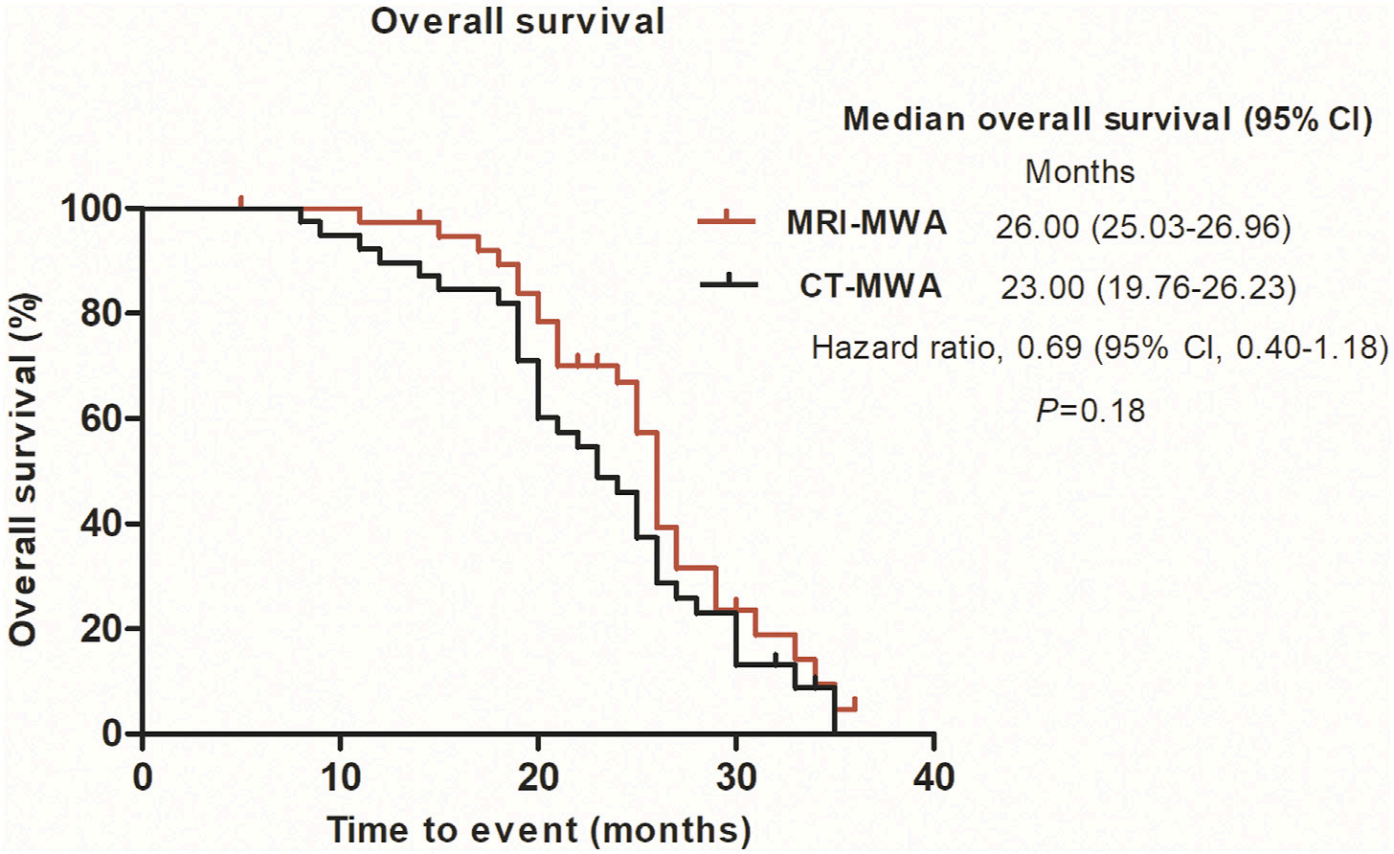
Graphs show Kaplan–Meier survival estimates for overall survival between MRI-MWA group and CT-MWA group.

The median OS was 26.0 months [95% CI 25.022–26.978] in the MRI group and 23.0 months [95% CI 18.646–27.354] in the CT group (*p* = 0.18) (Figure 6).

## Discussion

It is well-established that CT-guided microwave ablation provides a minimally invasive approach to eradicating tumors, especially for solid lung neoplasms (Wolf et al., 2008; Ko et al., 2016; Huang et al., 2020). The advantages of microwave ablation include higher intratumoral temperatures, the ability to use multiple applicators, more rapid and homogeneous ablations, and little influence of heat dispersion on blood circulation (Skinner et al., 1998; Stauffer et al., 2003; Wright et al., 2003; Shock et al., 2004). Although microwave ablation for lung cancer has achieved remarkable results, there are still some issues, such as inaccurate localization, subjective selection of ablation parameters, incomplete ablation, and excessive ablation, which directly affect the safety and effectiveness of this approach (Liang et al., 2009; Chen et al., 2020). MRI-guided MWA can compensate for the limitations of CT-guided ablation and has similar advantages to CT. Indeed, MRI-guided microwave ablation does not use ionizing radiation and provides high soft-tissue resolution, arbitrary orientation, and multi-parameter imaging for the ablation of liver, kidney, prostate, and other tumors (Lin et al., 2019; Faridi et al., 2020; Yang et al., 2020).

Our preliminary results show that MRI imaging yields superior accuracy, relative to CT, during ablation needle puncture. It is well-established that a fast sequence, such as the T1-TFE sequence, can distinguish anatomical structures for precise puncture. MRI can be used for arbitrary multidirectional imaging to ensure that the overall microwave antenna and the relationship between the microwave antenna and the lesion can be displayed during puncture, shortening the operation time and achieving a rapid, accurate puncture location. Moreover, the metal artifact produced by the ablation needle during CT can cover the ablation target. Importantly, magnetic resonance multisequence imaging and MR-functional imaging methods such as DWI, MRS, and PWI provide more accurate and abundant information than CT does for assessing the relationship between lung tumor lesions and surrounding tissues, cardiac great vessels, and the ablation boundary, making the puncture process simpler, safer, and more accurate (Biederer et al., 2012). In our study, the number of scans required for ablation-needle puncture in the MRI-MWA group was significantly less than in the CT-MWA group (7.62 ± 1.69 vs. 9.64 ± 2.14, *p* < 0.001). Although the average single-scan duration for MRI was slightly longer than for CT, the average times for antenna placement were comparable for the MRI-MWA group and the CT group (53.03 ± 11.29 min and 54.41 ± 12.32 min, *p* = 0.607). The total procedure duration was comparable between both groups (91.28 ± 16.69 min vs. 93.41 ± 16.03 min, *p* > 0.05). Based on the accuracy of MRI-guided localization and the application of rapid sequence, the overall scanning duration and operation duration were comparable to the CT-MWA group. Taken together, the aforementioned findings suggest that the overall efficiency of MRI-guided microwave ablation is consistent with that of CT-guided microwave ablation and yields less damage due to a significant reduction in the number of needle adjustments.

Indeed, the effect of ionizing radiation produced by CT scans, especially for tumor patients, should not be overstated (Pearce et al., 2012; Hu et al., 2016; Meulepas et al., 2019). Importantly, microwave ablation guided by magnetic resonance produces no ionizing radiation.

In a previous study, we demonstrated a significant difference between the ablation power and the actual ablation power, and it was challenging to accurately control the ablation range (Li et al., 2020). Excessively high temperatures may lead to carbonization of surrounding tissues, reduced ablation volume, or breakdown of the ablation needle due to the standing wave effect. If the ablation-zone temperature is too low, the tumor cannot be completely ablated. Therefore, real-time monitoring during ablation is important. CT is the primary imaging modality used to assess ablation results. Successful ablation is defined as a region of low attenuation covering the lesion area and a lack of contrast enhancement (Abtin et al., 2012; Iguchi et al., 2015; Ye et al., 2018; Ye et al., 2021). It has been shown that in well-demarcated ablation areas, necrosis is surrounded by a darker, patchy rim of acute inflammation, marked congestion, edema, and diffuse alveolar hemorrhage (Goldberg et al., 1995). However, these changes are not obvious on CT, especially in the marginal areas, and there is no guarantee that the potential residual foci near blood vessels could be completely removed. However, based on our team’s experience, ground-glass shadow after ablation is often confused with bleeding caused by mechanical lung-tissue injury during puncture in plain CT scanning. Thus, CT does not bring significant advantages during intraprocedural therapeutic evaluation (Harada et al., 2011). It is well-established that MRI is sensitive to changes in the water content of ablated lesions (Lazebnik et al., 2003; Lin et al., 2019). Importantly, high-contrast MRI provides high-definition images of soft tissue, clarifies the lesion’s scope, and distinguishes liquefied necrotic tissue from active tumor lesions. Current evidence suggests that MRI enables visualization of coagulated necrotic areas of 2–3 mm (Song et al., 2020). Multi-parameter imaging, such as tissue-diffusion and perfusion imaging, provides a more accurate evaluation of the therapeutic effect (Hayashida et al., 2006; Kamel et al., 2006; Ohira et al., 2009; Olin et al., 2019). Any azimuth fault can clearly display the boundary and formation process of ablation foci. Therefore, the parameters measured by MRI can be used to determine the ablation effect and endpoint to improve the local ablation-control rate, reduce tumor residue, and reduce the incidence of complications (Li et al., 2017).

In the present study, we quantified the maximum diameter, including the ablation area, of isointense signal intensity on T1WI and of high signal intensity on T2WI images, without using contrast enhancement. These findings suggest that, compared with CT, MRI can provide a more accurate real-time evaluation of MWA. T2WI can be used to evaluate the effect of WMA. In the present study, the mean PFS was 11 months in the MRI-MWA group and 9 months in the CT-MWA group, and the difference was significant (χ^2^ = 11. 237, *p* < 0.01), while no difference in OS was observed between the groups at 3-year follow-up (χ^2^ = 2.256, *p* = 0.132).

Although both groups had similar survival rates, higher PFS was observed in the MRI-MWA group, suggesting that these patients had a better quality of life. Nonetheless, it remains unclear whether MRI-guided MWA could improve the survival rate, and further studies with longer-term follow-ups are warranted.

Compared with the literature, PFS was significantly improved between the experimental and control groups in this study, which may be related to albumin-bound paclitaxel (Jung et al., 2020; Xu et al., 2021). Nab-paclitaxel is a nanoparticle-sized anti-tumor drug formed from paclitaxel and albumin (West et al., 2019). It has been found that nab-paclitaxel can be used instead of regular paclitaxel to reduce hypersensitivity reactions in the treatment of advanced breast cancer (Schmid et al., 2018; Emens et al., 2021). In addition, nab-paclitaxel has targeting properties which, combined with the natural transport mechanism of albumin, allow paclitaxel to act on tumor tissues and achieve higher drug levels within a short period, thereby achieving better anti-tumor effects. Its main mechanism of action is similar to paclitaxel—promoting microtubulin polymerization and hindering microtubulin depolymerization—and thus it inhibits tumor cell proliferation and growth (Yardley, 2013). Moreover, overwhelming evidence has substantiated that nab-paclitaxel is effective as the first-line treatment of advanced NSCLC. A large phase-III clinical study showed that nab-paclitaxel combined with carboplatin was cost-effective, compared to paclitaxel injection combined with carboplatin, for the first-line treatment of advanced NSCLC (Jotte et al., 2020).

Although survival was similar between the two groups in this study, the higher PFS rate in the MRI-MWA group suggested that its patients had a better quality of life. However, it remains unclear whether MRI-guided microwave ablation could improve the survival rate; this uncertainty emphasizes the need for further studies with longer follow-ups to validate this hypothesis.

Similar to percutaneous ablation therapies, MRI-guided microwave ablation is associated with common complications such as hemorrhage, pleural effusion, pneumothorax, and cavitation of the ablation zone (Chen et al., 2020). Other complications, such as pulmonary-artery aneurysms and postprocedural pneumonia, are rare and were not observed in our study. Moreover, we found no difference in the incidence of complications, although the incidence of ablation-zone cavitation was slightly higher in the CT-MWA group than in the MRI-MWA group, which may be related to the longer ablation time (Carrafiello et al., 2012; Zheng et al., 2014; Welch et al., 2015). Although cavitation of the ablation zone is usually clinically insignificant, rupture may occur, leading to pneumothorax and bleeding. Cavities may also serve as scaffolds for fungal colonization. Precautions to minimize these risks should be taken whenever possible. In most cases, patients in the MRI-WMA group did not experience any symptoms, with only one experiencing an increased heart rate (90 bpm) that was resolved by manual aspiration.

The present study had some limitations, including its small sample size and the retrospective nature of the data obtained for the CT-MWA group. Moreover, our findings were based on data from a single center, emphasizing the need for multi-center studies. In this study, ablations were performed by experienced interventionists; it remains unclear whether the same benefits in accuracy and time would be observed with less-experienced radiologists. Moreover, the management of patients (i.e., whether they received targeted therapy, immunotherapy, or systemic chemotherapy) after local tumor progression was not assessed in the 3-year follow-up, but this will be carried out during follow-up analysis. Additionally, the quality of life and other measures of patient experience of the treatment were not directly studied and should be considered in the future to truly reflect the value of MRI-guided microwave ablation for the treatment of NSCLC. Indeed, the follow-up period (3 years) was relatively short. A longer follow-up period may reveal differences in survival rates between the two groups.

## Conclusion

Overall, the results of our matched-cohort study suggest that MRI-guided percutaneous ablation has significant prospects for the treatment of lung tumors as a feasible, safe, effective, and minimally invasive approach and provides a satisfactory outcome.

## Data Availability

The original contributions presented in the study are included in the article/Supplementary Material; further inquiries can be directed to the corresponding author.
